# Coastal land uplift and intensified land-use influence seagrass carbon and nitrogen sink capacity over millennial timescales

**DOI:** 10.1038/s41598-026-54674-y

**Published:** 2026-05-26

**Authors:** Martin Dahl, Sara Braun, Maria E. Asplund, Mats Björk, Joeri Kaal, Hans W. Linderholm, Elinor Andrén, Thomas Andrén, Žilvinas Ežerinskis, Madhavu Vidya Faizal, Sara C. Forsberg, Andrius Garbaras, Malin E. Kylander, Pere Masqué, Miguel A. Mateo, Justina Šapolaitė, Oscar Serrano, J. Robin Svensson, Olena Vinogradova, Martin Gullström

**Affiliations:** 1 School of Natural Sciences, Technology and Environmental Studies, Södertörn University, Huddinge, Sweden; 2 Department of Biological and Environmental Sciences, University of Gothenburg, Kristineberg, Fiskebäckskil, Sweden; 3 Department of Ecology, Environment and Plant Sciences, Stockholm University, Stockholm, Sweden; 4 Pyrolyscience, Santiago de Compostela, Spain; 5 Regional Climate Group, Department of Earth Sciences, University of Gothenburg, Gothenburg, Sweden; 6 Center for Physical Sciences and Technology, Vilnius, Lithuania; 7 Department of Geological Sciences, Stockholm University, Stockholm, Sweden; 8 Centre for Marine Ecosystems Research, School of Natural Sciences, Edith Cowan University, Joondalup, WA Australia; 9 International Atomic Energy Agency, Principality of Monaco, Monaco; 10 Centro de Estudios Avanzados de Blanes, Consejo Superior de Investigaciones Científicas (CEAB-CSIC), Blanes, Spain; 11 Department of Marine Sciences, University of Gothenburg, Gothenburg, Sweden

**Keywords:** Blue carbon, Baltic sea, Zostera marina, Land-use, Coastal change, Paleoreconstruction, Climate sciences, Ecology, Ecology, Environmental sciences, Ocean sciences

## Abstract

Seagrass meadows store sedimentary carbon and nitrogen and thus play an important role in climate change mitigation and nutrient retention globally. Here we reconstruct the impact of land-use and coastal land uplift on millennial–scale seagrass (*Zostera marina*) blue carbon and nitrogen accumulation in the Baltic Sea based on a multiproxy paleoreconstruction approach. We show that increased landscape clearance resulted in higher terrestrial runoff and increased accumulation of carbon and nitrogen, whereas land uplift led to hydrodynamically sheltered environments facilitating seagrass colonization, occurring 1700 and > 4000 years ago. The establishment of the seagrass meadows resulted in enhancement of the quality of carbon and nitrogen stocks with increased lignin contents. Seagrass establishment also likely supported export of organic matter to surrounding unvegetated areas and thereby contributed to carbon and nitrogen accumulation beyond habitat boundaries. The findings show that the long-term carbon and nitrogen accumulation rates and permanence are shaped by land cultivation and geomorphological changes and highlights the importance of seagrass conservation for maintaining thousands of years of sedimentary carbon and nitrogen storage.

**Supplementary Information:**

The online version contains supplementary material available at 10.1038/s41598-026-54674-y.

## Introduction

Seagrass meadows can effectively accumulate carbon and nitrogen in the underlying sediment over centennial to millennial timescales^1–3^. The permanence of carbon and nitrogen sinks over large timescales is important in mitigating climate change and eutrophication through atmospheric carbon dioxide removal and nutrient filtration. Seagrasses are ecological engineers that can transform the underlying sediment by increasing the accumulation of organic matter through high internal meadow productivity (autochthonous production) and the trapping of allochthonous organic matter from adjacent ecosystems^4^. Substrate stabilization by the seagrass plants (i.e., by roots and rhizomes) and biogeochemical processes lead to increased sedimentation rates, and the accumulation of fine grain-sized particles and biological material that subsequently reduce oxygen levels, creating conditions that favor the preservation of organic matter^5–7^. Such biogeochemical conditions have been compared to the pedogenic processes in terrestrial soils with histic properties (water-saturated and organic-rich soils)^8^. Under favorable environmental conditions, the accumulation of organic-rich sediments can create meters-thick sediment layers that can persist for thousands of years^2,9^.

Eelgrass (*Zostera marina* L.), the seagrass species with the largest distribution range globally, is mainly found in sheltered or semi-sheltered areas across temperate regions of the Northern Hemisphere^10,11^. In cold-temperate regions, carbon and nitrogen stocks in *Z. marina* meadows have been widely studied (e.g.,^12–15^) as well as short-term (decades to century) accumulation rates^3,16–18^. There is, however, a lack of studies that have quantified the permanence of these carbon and nitrogen sinks over millennial time scales^19^. In the Baltic Sea specifically, *Z. marina* is widespread across Kattegat (mainly growing in monospecific meadows) and the Baltic Proper (commonly growing mixed with other rooted vegetation, e.g., *Stuckenia* sp. and *Zannichellia* sp*.*^20^), but has declined from historical area and depth distribution due to eutrophication^21^. Several studies have assessed *Z. marina* carbon stocks in the Baltic Sea (e.g., ^22–24^), showing that the levels are lower compared to other regions^15,25,26^. The nitrogen retention capacity and the long-term accumulation of carbon rates of Baltic *Z. marina* meadows, both short– or long timescales, remain poorly known.

Environmental conditions and climate have varied greatly during the Holocene (the last ~ 11,700 years) in the Baltic Sea region, with major geomorphological transformations in the coastal zone following the retreat of the Scandinavian Ice Sheet at the onset of Holocene^27–29^. The melting of the ice sheet led to major isostatic land up lift and eustatic sea level changes that caused large shoreline oscillations in the early to mid-Holocene^30–32^ with shoreline displacement up to 285 m above present sea level^33^. Land uplift still continues in the northern Baltic Sea while the southern Baltic basin experiences land subsidence^34^. The effect of land uplift will likely be reduced in the future from the ongoing sea level rise^35,36^. The coastal geomorphological changes during Holocene resulted in the creation of a complex coastal archipelago with islands, islets, skerries and embayments, including hydrodynamically sheltered and shallow environments suitable for seagrass colonization^19^.

The Baltic Sea coastal region has been populated for thousands of years, with agricultural- and other land-use practices that have modified the landscape. In Southern Sweden, human-induced landscape openness begun already 2000–1000 BCE (Before Common Era) with the increase of grazing and farming practices^37,38^, and the level of open land vegetation has since varied depending on population dynamics^37^ with rapid changes occurring during the last two centuries^39^. Land clearance can enhance the flow of materials from land to sea and thereby potentially influence the carbon dynamics of coastal vegetated habitats^40,41^. Deforestation can accelerate carbon export and burial to nearby coastal habitats^42^, but at the same time excessive sediment loads can increase turbidity, pollution and eutrophication that can compromise seagrass growth, and thereby reduce the carbon sequestration capacity of seagrass meadows^43–45^. Stable isotopic signatures of carbon (δ^13^C) and nitrogen (δ^15^N) are useful in tracing coastal carbon and nitrogen sources, respectively. More negative δ^13^C values (around -30 to -25‰) indicate terrestrial material, while less negative values (about -20 to -10‰) characterize marine sources^46,47^. An enrichment of δ^15^N is commonly used to identify anthropogenic nitrogen inputs, from e.g. agricultural runoff or wastewater discharge^48^, as a proxy for the anthropogenic influence on coastal sediments^49,50^. Other tracers, such as biomarkers, eDNA and biomolecular compounds, can also provide information on the origin and sources of organic matter in coastal sediments at various spatial and temporal scales^51–54^. For example, specific lignin products and derivates originated from seagrass plant material have been used to estimate seagrass input to the sedimentary OC pool^55–58^. The source composition influences the quality of the organic matter (i.e., the degradation potential) being accumulated and thus the permanence of the carbon and nitrogen sinks^59^ as e.g., organic matter high in lignin and other refractory compounds derived from seagrass plants or terrestrial vascular plants are more resilient to degradation^60–63^.

**Fig. 1:**
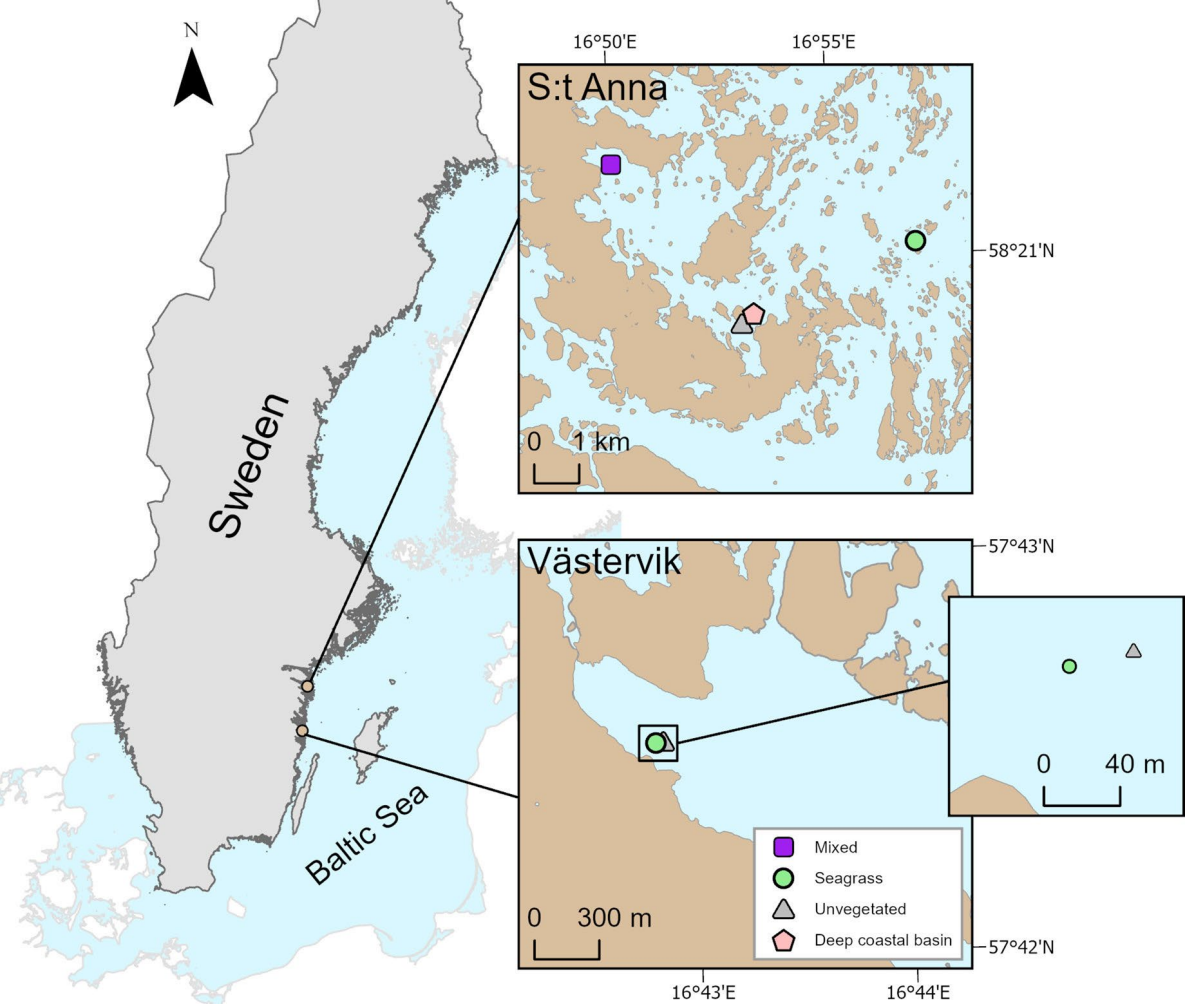
Study area and sampling locations in S:t Anna (four sites) and Västervik (two sites) on the Swedish east coast in the Baltic Proper. The S:t Anna study location is situated within a larger archipelago area with scattered islands and islets, while the Västervik location is situated within an embayment. All sampling sites were found in relatively sheltered environments with a relative fetch of 0.52–0.78 km, but in S:t Anna, the study sites (except for the mixed seagrass meadow) were situated in the mid- to outer parts of the archipelago while the Västervik sites were located close to mainland. In S:t Anna, the mixed seagrass meadow (sampled at 3 m water depth) comprised of *Z. marina* and other rooted vegetation (i.e., *Ceratophyllum* sp., *Myriophyllum* sp., *Stuckenia* sp. and *Zannichellia palustris*), while the other two seagrass meadows in S:t Anna (5 m) and Västervik (5.4 m) were monospecific *Z. marina* meadows. The unvegetated sites were located slightly deeper, at 6 m in S:t Anna and at 7.3 m in Västervik. An even deeper coastal accumulation basin site in S:t Anna (17 m) was included to evaluate the potential export of seagrass-derived organic matter to deeper habitats in the coastal zone.

In this study, we assessed the influence of land-use (i.e., increased areas of cropland and grassland) and coastal change (i.e., land uplift) on seagrass sedimentary organic carbon (OC) and total nitrogen (TN) contents, and the quality of organic matter (i.e., lignin content) over the Holocene in the southwestern Baltic Sea using multiproxy paleoreconstruction methods (Fig. 1). We also identified the historical onset of seagrass colonization and associated biogeochemical changes of the sediment using lignin products derived from seagrass plant biomass and stable isotope signatures. In addition, we described and compared the seagrass meadows’ OC and TN storage capacity in relation to adjacent unvegetated sediments, including the potential export of seagrass-derived organic matter to surrounding habitats.

## Results

### Sediment age models

All cores showed decay of excess ^210^Pb that allowed estimating mass and sediment accumulation rates (MAR and SAR) from early to mid–1900 CE until present (Fig. S1). There was mixing of the sediment in the seagrass meadow at S:t Anna from 0 to 2 cm and the MAR and SAR were extrapolated over these depths. The MAR and SAR in the Västervik sites should be seen as upper limits due to possible diffusion of excess ^210^Pb. The seagrass and unvegetated cores in S:t Anna showed an apparent hiatus (break in sediment continuity) at 10–13 cm sediment depth and therefore no radiocarbon age modelling was performed in these two cores. The mean (± SD) calibrated radiocarbon for the oldest section of the cores in Västervik were ~ 4100 ± 50 BCE (41 cm) for the *Zostera* meadow and ~ 4200 ± 40 BCE (39 cm) for the unvegetated site. In S:t Anna, the mean (± SD) calibrated radiocarbon age for the oldest section of the mixed meadow core was 1930 ± 60 years BCE (83 cm) (Figs. S2 and S3; Table S1). The mean sediment accumulation rates obtained with ^210^Pb allowed estimating the ages (based on the Constant Rate of Supply [CRS] model) for the seagrass and unvegetated sites in S:t Anna until 1919 ± 4 years CE (9 cm) and 1960 ± 4 years CE (6 cm), respectively.

### Sediment biogeochemical properties in seagrass and unvegetated sediments

**Fig. 2:**
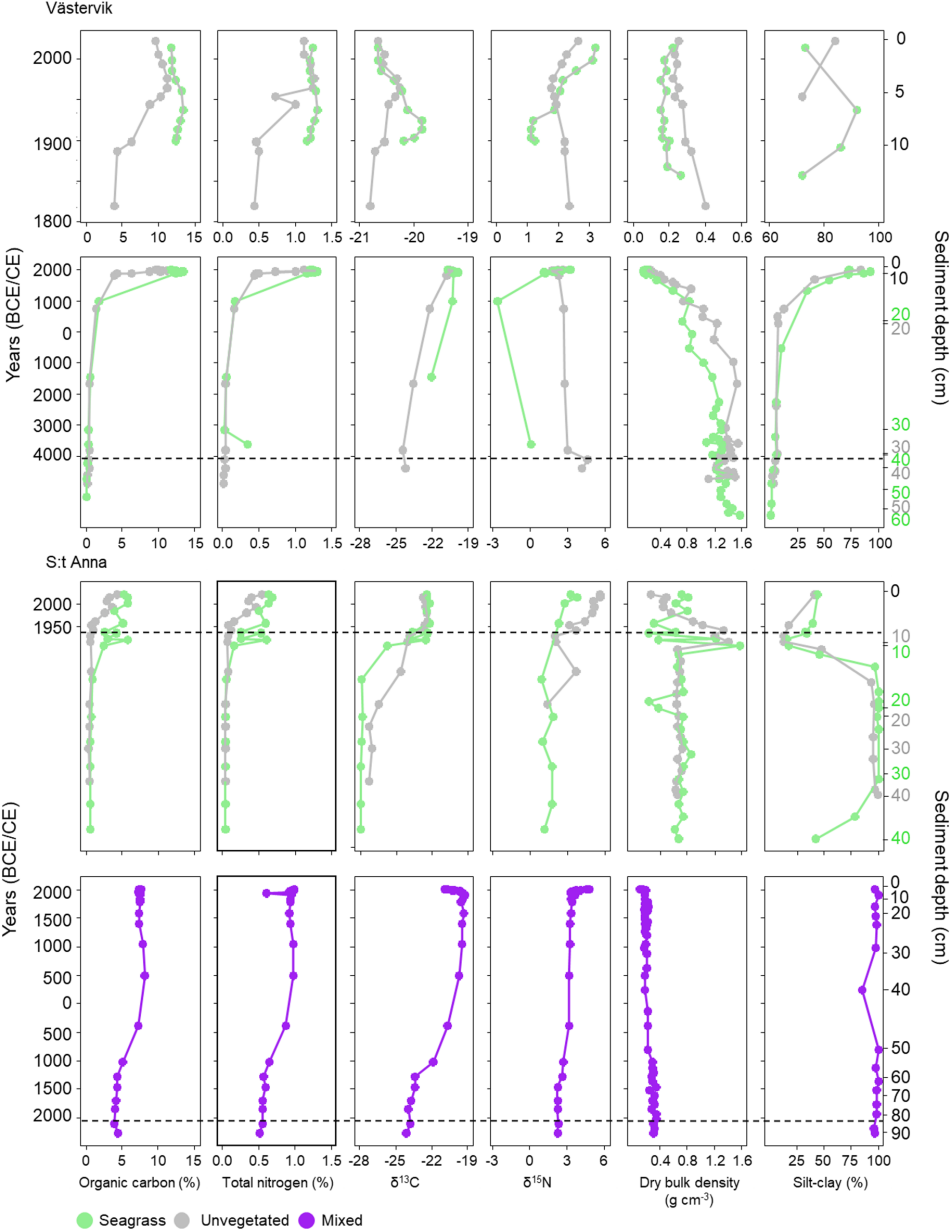
Sediment downcore trends for organic carbon (OC), total nitrogen (TN), stable isotopes (δ^13^C and δ^15^N), dry bulk density (DBD) and silt–clay fractions (< 63 µm). For Västervik, the top panel is zoomed in for better visualization of the last ~ 200 years; note the different x-axis scale in this panel. The dashed lines indicate the maximum age of the age-depth models. In the seagrass and unvegetated sites of S:t Anna, there was an apparent hiatus at 10–13 cm and therefore no radiocarbon dating was performed in these cores.

The dry bulk density (DBD) in Västervik was stable at the bottom of the core and decreased gradually towards the surface from 350 and 270 CE (20 cm) for the seagrass and unvegetated sites, respectively (Fig. 2). In S:t Anna, the DBD was stable from the bottom of the core to 14–15 cm for the seagrass and unvegetated sites. From 14–15 cm to 7–10 cm, large fluctuations in DBD followed by more stabilized values towards the sediment surface occurred (Fig. 2). In the *Z. marina* meadow, the DBD remained constant with a mean (± SD) of 0.22 ± 0.06 g cm^-3^ for the entire sediment core. The bottom of both cores from Västervik contained predominantly sand (Fig. 2) with low mud (silt–clay fractions < 63 µm) content (< 10%), while from around 1300 to 1700 CE (13 and 17 cm), the silt–clay content increased towards the sediment surface reaching 84 and 92% for the unvegetated and seagrass sites, respectively. The seagrass and unvegetated cores in S:t Anna were made up of post-glacial clays in the bottom of the cores (Fig. S2) and were almost entirely fine grain-sized particles (Fig. 2). The silt–clay content decreased in both these cores at 13 cm, while at 10 to 8 cm and towards the sediment surface, we observed a clear increase to more than 40%. In the mixed meadow, the sediment was homogenous and comprised of mud with high OC-content throughout the sediment profile (Figs. S1 and 2).

**Fig. 3:**
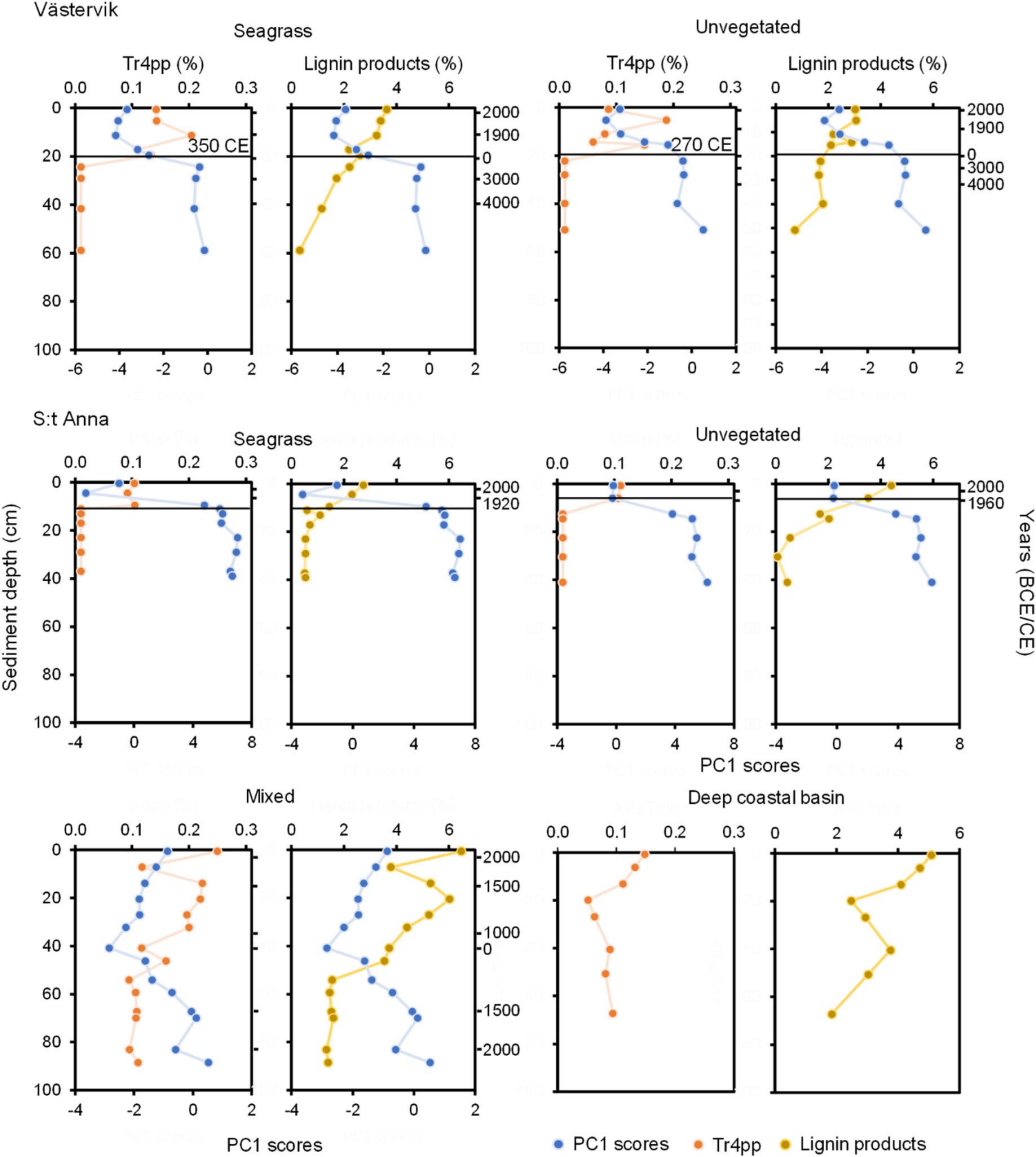
Sediment downcore trends showing PC1 scores, tr4pp and lignin products. The solid lines show the first appearance of tr4pp in the sediment records, which is used as a proxy for seagrass colonization with the dates showing the corresponding age at that sediment depth. In the seagrass and unvegetated cores from S:t Anna, the seagrass colonization occurred prior to the maximum age of the ^210^Pb-dating and therefore the age of colonization could not be established.

The OC and TN contents increased towards the surface of the sediment profiles for all cores (Fig. 2). In Västervik, the average OC and TN contents were 9–28-fold higher from 1900 CE (11 and 8 cm) to present for the seagrass and unvegetated sites in comparison to 4300–4000 BCE (38–39 cm) to 1900 CE (Fig. 2; Table S2). The seagrass and unvegetated cores in S:t Anna showed a similar, albeit lower, average increase in OC and TN contents in the surface layers, with a 7- to 14-fold increase in the seagrass meadow at 13 cm and a 5- to sixfold increase from ~ 1950 CE (9 cm) in the unvegetated site (Fig. 2). In the mixed site, a twofold increase in OC and TN content occurred around 380 BCE (46 cm) and remained stable to present (Fig. 2). In the upper 10–20 cm sediment depths, seagrass sediment (excluding the mixed meadow in S:t Anna) had significantly higher OC and TN content in comparison to unvegetated sites (*p* < 0.001; Fig. S3). The stable carbon isotope values, used as an indicator of organic matter sources in the sediment, in Västervik increased moderately towards the surface of the sediment, with less negative values (from -21 to -20 ‰) in the more recent deposits in the seagrass (from 950 CE, 18 cm) and unvegetated sites (from 1820 CE, 11 cm). In S:t Anna, a shift towards less negative values (from -26 to -22 ‰) was observed at 13 cm sediment depth in the seagrass meadow, while a more gradual increase from -25 to -22 ‰ was seen around 20 cm sediment depth. In the mixed meadow, there was a shift from -24 to -19 ‰ at ~ 1300 BCE (59 cm) followed by a gradual increase until ~ 1800 CE (11 cm) when the δ^13^C decreased again towards the surface. The nitrogen stable isotope (δ^15^N) values increased in the surface sediment for all cores except the cores in the unvegetated site in Västervik, which had its highest δ^15^N value (4.2 ‰) at 4100 BCE (34 cm). The seagrass site in Västervik reached the highest δ^15^N values (3.1–3.2 ‰) at the sediment surface (2000–2010 CE, 1–2 cm). In S:t Anna, the seagrass and unvegetated sites showed an increase in δ^15^N from around the 1950s (at 6 and 9 cm, respectively), with the highest values found in the unvegetated site (5.6‰) measured in the top 2 cm (2010–2020 CE) (Fig. 3). In the mixed site, the δ^15^N remained stable with values of ~ 3‰ from the bottom of the sediment core until the ~ 1960s when the values increased to a maximum of 4.6‰ (Fig. 2).

PCA was applied to determine the general trend of the biogeochemical variables of the sediment record and for assessing common shifts in the sedimentary variables combined. The two first principal components (PCs) in the PCA explained 79% of the variation of which PC1 described 47% and PC2 32% of the variance (Table 1). PC1 had positive loadings (> 0.7) for K, Ti, Si, Mn, Ca, Fe and Rb (see Table 1 caption for full names of the chemical elements), and negative loadings (< -0.7) for Cl, Ni, Br and δ^13^C. PC2 showed positive loadings for Zn, silt–clay, TN and OC, and negative loadings for DBD and Sr (Table 1). Change Point modelling (CPM) was applied for PC1 and PC2 scores for each core to decipher common shifts among the biogeochemical variables. For the seagrass site in Västervik, PC1 change points were identified at 1900 CE (13 cm), 1000 BCE (23 cm) and 48 cm (no date), and for PC2 at 1900 CE (13 cm), 500 BCE (22 cm) and 3500 BCE (33 cm) (Fig. S5). The unvegetated site in Västervik had change points identified for PC1 scores at 1890 CE (9 cm), 250 BCE (21 cm) and 43 cm (no date), and for PC2 at 1890 CE (9 cm), 1700 BCE (23 cm) and 42 cm (no date) (Fig. S5). Change points for the seagrass core at S:t Anna were identified at 10 and 8 cm for PC1 and PC2, respectively, and for the unvegetated site at 13 cm, and 9 and 15 cm for PC1 and PC2, respectively (Fig. S5). In the mixed meadow, one change point was identified for PC1 at 1500 BCE (65 cm) (Fig. S5).

**Table 1:** Factor loadings of the Principal Component Analysis (PCA) for PC1 and PC2 based on the biogeochemical variables for all five cores combined. Bold values show factor loadings > 0.7 and < -0.7. K = Potassium, Ti = Titanium, Si = Silicon, Mn = Manganese, Ca = Calcium , Fe = Iron, Rb = Rubidium, Zn = Zinc, DBD = dry bulk density, Sr = Strontium, Zr = Zirconium, S = Sulfur, TN = total nitrogen, OC = organic carbon, Cl = Chlorine, Ni = Nickel, Br = Bromine.

Biogeochemical variables	PC1 (47%)	PC2 (32%)
K	**0.96**	− 0.12
Ti	**0.87**	0.43
Si	**0.84**	− 0.44
Mn	**0.82**	0.53
Ca	**0.78**	− 0.52
Fe	**0.71**	0.67
Rb	**0.70**	− 0.11
Zn	0.50	**0.75**
DBD	0.28	− **0.91**
Silt–clay	0.11	**0.96**
Sr	− 0.04	− **0.97**
δ^15^N	− 0.21	− 0.17
Zr	− 0.47	− 0.31
S	− 0.65	− 0.41
TN	− 0.66	**0.72**
OC	− 0.67	**0.70**
Cl	− **0.74**	− 0.37
Ni	− **0.86**	− 0.13
Br	− **0.87**	0.36
δ^13^C	− **0.93**	0.18

### Carbon and nitrogen accumulation rates

In all cores where comparisons could be made (i.e., excluding the seagrass and unvegetated sites in S:t Anna), the mean levels of MAR and SAR were between 0.3- and fivefold higher in the recent short-term accumulation (~ 100 years) compared to the long-term accumulation (~ 1000 years) (Table 2). In Västervik, the short-term (~ 100 years) organic carbon accumulation rate (CAR) and total nitrogen accumulation rate (NAR) were 4- and fivefold higher in both the seagrass- and currently unvegetated sites compared to the levels on a long-term time scale (~ 1000 years), respectively. In the mixed meadow in S:t Anna, the short-term CAR and NAR were 3- and twofold higher compared to the long-term time scale, respectively (Table 2). The C/N ratios were estimated as a proxy for OC quality, and the results obtained showed relatively stable trends over short- and long-term time scales at Västervik sites (ranging 9.6–10.3), and in the mixed meadow at S:t Anna (ranging between 7.7 and 7.9). The C/N ratios of the seagrass and unvegetated sites in S:t Anna varied from 8.1 to 8.5 (Table S2).

**Table 2:** Mass (MAR), sediment (SAR), organic carbon (CAR) and total nitrogen (NAR) accumulation rates for three different time intervals, i.e., short term based on estimates from ^210^Pb dating (~ 100 years, ranging from 75 to 125 years), long term (~ 1000 years) and since the colonization of seagrass (~ 1700 years) at Västervik.

		Time interval	MAR (g cm ^-2^ yr ^–1^)	SAR (mm yr ^-1^)	CAR (g OC m ^-2^ yr ^-1^)	NAR (g N m ^-2^ yr ^–1^)
Västervik	Seagrass	Short term	0.016 ± 0.0004	0.92 ± 0.02	20.1 ± 0.6	2.0 ± 0.1
		Long term	0.004 ± 0.001	0.18 ± 0.06	4.8 ± 1.3	0.5 ± 0.1
		Seagrass colonization	0.004 ± 0.001	0.12 ± 0.04	3.2 ± 1.0	0.3 ± 0.1
	Unvegetated	Short term	0.017 ± 0.001	0.67 ± 0.03	17.0 ± 0.8	1.8 ± 0.1
		Long term	0.007 ± 0.0004	0.16 ± 0.01	4.3 ± 0.3	0.4 ± 0.03
S:t Anna	Seagrass	Short term	0.041 ± 0.002	0.98 ± 0.04	21.0 ± 0.9	2.5 ± 0.1
	Unvegetated	Short term	0.043 ± 0.002	0.74 ± 0.03	9.3 ± 0.4	1.1 ± 0.1
	Mixed	Short term	0.018 ± 0.002	1.09 ± 0.10	13.2 ± 1.2	1.7 ± 0.2
		Long term	0.005 ± 0.001	0.32 ± 0.05	3.7 ± 0.5	0.5 ± 0.1

### History of seagrass colonization periods

The relative proportions of different lignin products, i.e., 4-vinylphenol, *trans*-4-propenylphenol (tr4pp), guaiacols and syringols, were used to decipher seagrass presence/absence within the sediment records. In particular, tr4pp was identified as a specific marker of the relatively stable seagrass lignin. The first appearance of seagrass-derived organic matter in Västervik was identified in the seagrass site around 350 CE (20 cm). The unvegetated site in Västervik showed similar proportion of lignin products as the seagrass site and presence of tr4pp from 270 CE (20 cm) (Fig. 3). There was also a shift into less negative δ^13^C values seen in the cores (from around –23–24 to –20‰), indicating a temporal change in organic matter source composition. Since the colonization of seagrass in Västervik, 4500 g OC m^-2^ and 440 g TN m^-2^ have accumulated. In S:t Anna, the seagrass and unvegetated sites showed similar trends in content of tr4pp and lignin products. The seagrass site showed the first occurrence of tr4pp at 10 cm, while in the unvegetated site it was first observed at 6 cm (from ~ 1980 CE). Presence of tr4pp and lignin products was seen throughout the entire sediment profile in the mixed meadow, indicating that the site had been vegetated at least ~ 4000 years, with an increase from around 1120 BCE (55 cm sediment depth) towards the sediment surface. The accumulated OC and TN over the last ~ 4000 years was estimated to 2580 g OC m^-2^ and 330 g TN m^-2^. The deep coastal basin site also showed tr4pp and lignin products along the entire sediment profile with an increase at 20 cm (no date) sediment depth (Fig. 3).

### Influence of terrestrial landscape openness and land uplift on sedimentary carbon and nitrogen contents

**Fig. 4:**
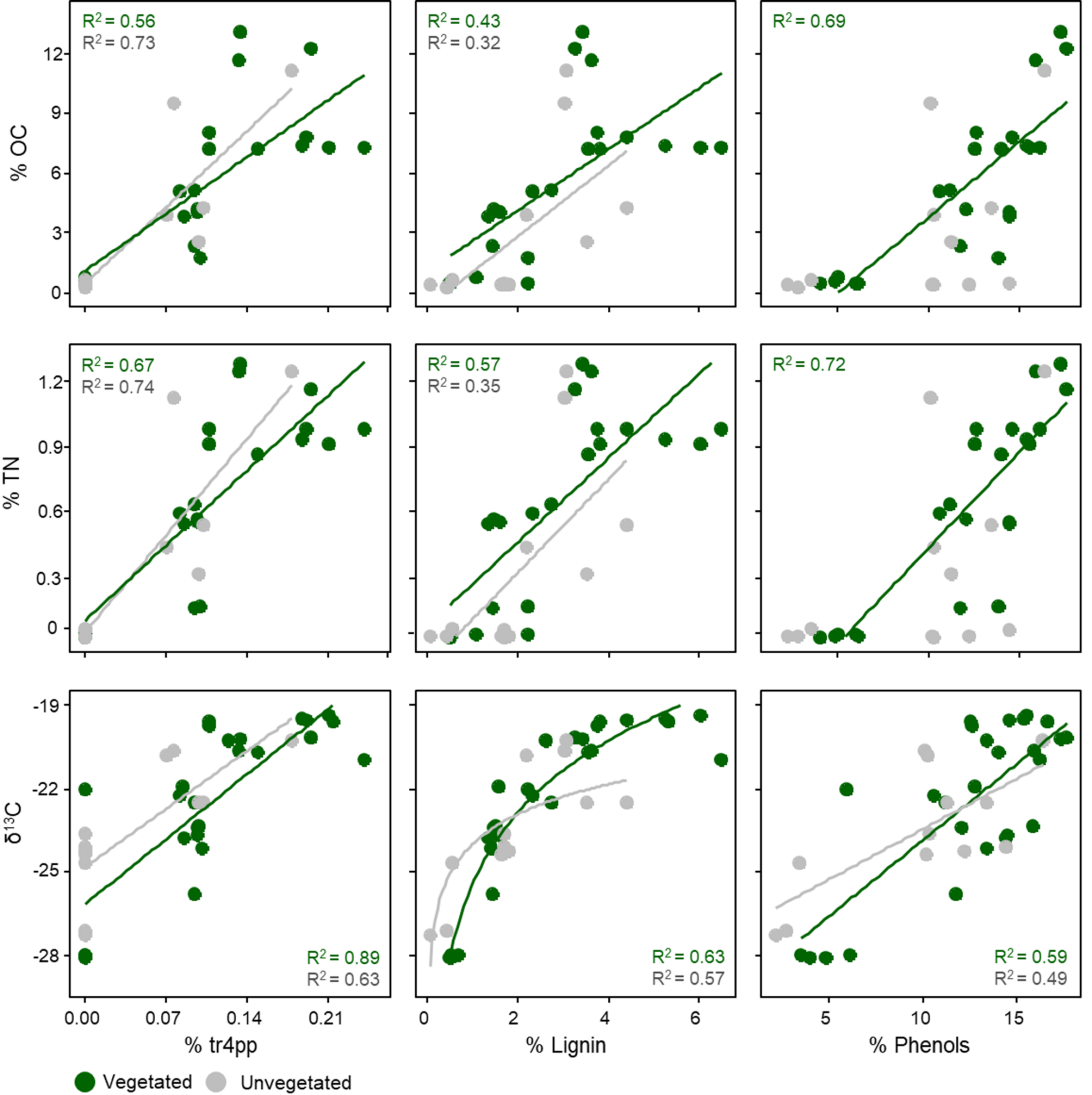
Linear regression analysis showing pairwise relationships between various carbon and nitrogen variables, (i.e. organic carbon (OC), total nitrogen (TN) and δ^13^C) and organic matter quality variables (i.e., %tr4pp [*trans* 4-propenylphenol], %lignin and %phenols) for vegetated (monospecific and mixed *Z. marina* meadows) (green dots) and unvegetated sites (grey dots) in S:t Anna and Västervik. Note that the δ^13^C–%lignin regression is based on log transformation.

**Fig. 5:**
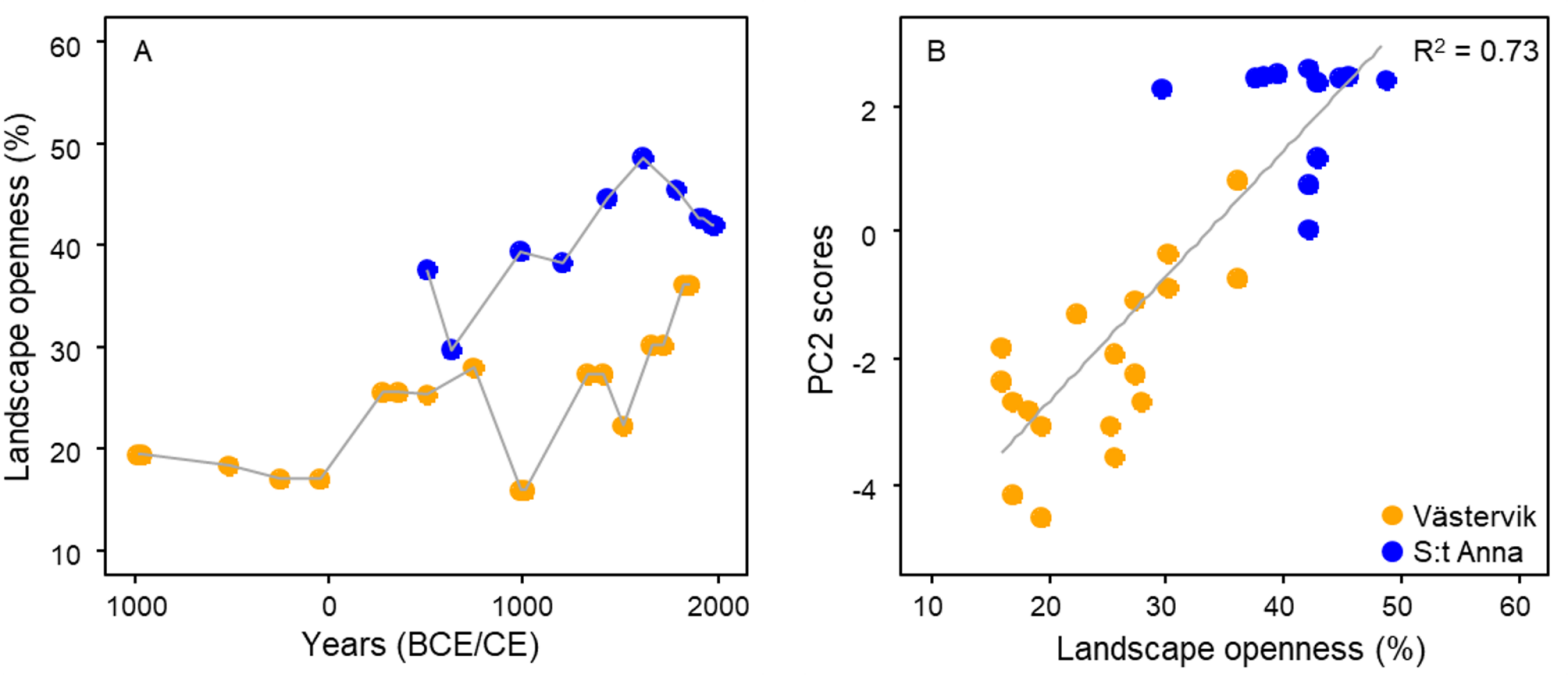
Terrestrial landscape openness (i.e., % of grassland and cropland) over time (**A**) and relationship between PC2 scores and % landscape openness based on linear regression analysis (**B**). Landscape openness is defined as the proportion (%) of open vegetation (i.e., cropland and grassland) based on high-resolution pollen records (Vinogradova et al.^64^; unpublished). The land-use models include the last ~ 3,000 years in Västervik (orange) and ~ 1,500 years in S:t Anna (blue).

Tr4pp was positively correlated to OC and TN in vegetated- (including both monospecific and mixed *Z. marina* meadows) and unvegetated sediments (*p* < 0.001; Fig. 4). The total proportion of lignin products was also positively correlated to OC and TN in vegetated- (*p* < 0.001) and unvegetated sediments (*p* < 0.05; Fig. 4). Other phenols (phenol and alkylphenols), probably mainly from *p*-hydroxyphenyl-type lignin and/or degraded lignin, were strongly positively correlated to OC and TN in vegetated sediment (*p* < 0.001), whereas no significant correlations were observed in unvegetated sediments (Fig. 5). Furthermore, lignin, tr4pp and phenols were all positively correlated with δ^13^C in both seagrass- (*p* < 0.001) and unvegetated sediments (*p* < 0.01) (Fig. 4).

**Fig. 6:**
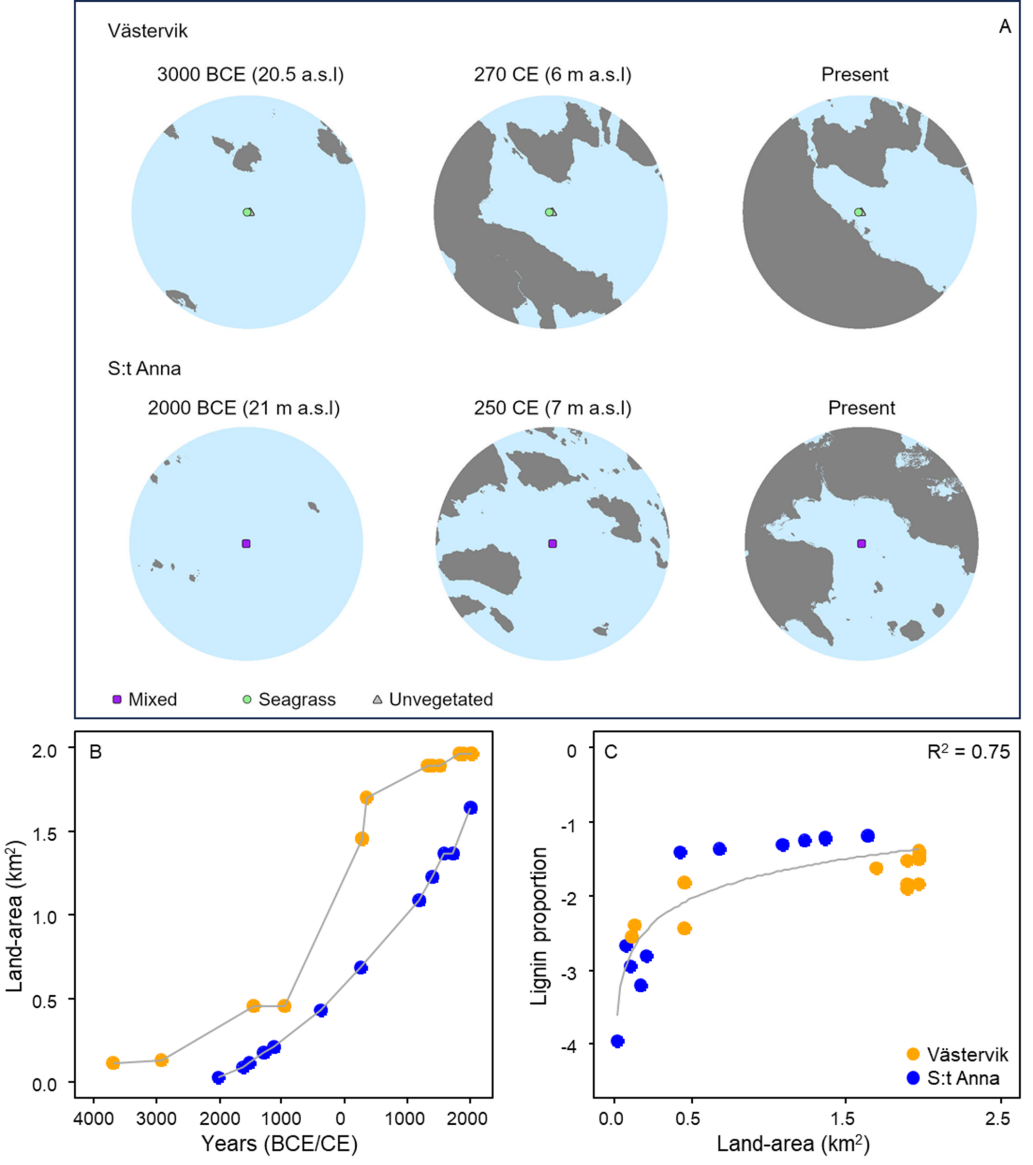
Paleogeographical maps in buffer zones at three different time periods in Västervik and S:t Anna (**A**), land area (km^2^) increase over time following land uplift reconstructions (**B**), and relationship between lignin content and land area at Västervik (orange) and S:t Anna (blue) (**C**). Note that lignin proportion was standardized using reciprocal transformation to account for different lignin proportion levels in the two sites.

There were positive correlations between terrestrial landscape openness (i.e., the proportion of crop- and grassland in relation to forested and wetland habitats) and PC2 scores (closely associated to silt–clay fractions, DBD and OC and TN contents; Table 1) (*p* < 0.001; Fig. 5), as well as between land uplift (i.e., the amount of land area in buffer zones around sampling sites) and lignin proportion (*p* < 0.001) for seagrass and unvegetated sediments (Fig. 6).

## Discussion

Land-use intensity coupled with land uplift and geomorphic changes influenced the rate and permanence of the seagrass OC and TN sinks over the past millennia in S:t Anna and Västervik, and hence shaped the coastal zone’s capacity for climate change mitigation and adaptation, and nutrient filtration. The positive relationship with land uplift also highlights that future sea level rise may arguably have a negative impact on the seagrass carbon and nitrogen sink capacity following land subsidence, which is already occurring in the southern Baltic Sea^34^, and potentially induce less favorable conditions for seagrass meadow establishment and integrity. Seagrass colonization and associated changes in the sedimentary biogeochemical properties increased OC and TN storage levels in comparison to adjacent unvegetated areas, showing the high capacity of seagrass meadows to accumulate organic matter. Furthermore, the results showed that seagrass colonization likely supported the export and storage of seagrass-derived organic matter to adjacent shallow and deep unvegetated habitats, which highlights the contribution of seagrass meadows to climate change mitigation beyond their habitats.

### The influence of land-use changes

Human land-use has been ongoing for thousands of years, which has transformed ecological functions and processes of the earth’s ecosystesms^65^. In our study sites, shifts in the area of open vegetation (i.e., grassland and cropland) following changes in land-use over the last ~ 3,000 years caused enhanced soil erosion and land runoff to the coastal zone, with fluxes of terrigenous material rich in fine grain-sized particles and organic matter that increased the OC and TN storage levels. The enhancement of land-sea transport of organic matter has previously been reported in seagrass meadows following disturbances^42,44,66^, which in turn led to increased OC storage levels in seagrass sediment^67,68^.

The Västervik sites showed marked changes in sediment biogeochemistry by ~ 1900 CE based on Change Point modelling (CPM) (Fig. S5), with increasing OC and TN contents from around 1900 until the 1970s that could be associated to the modern transformation of the landscape and associated run-off, e.g., the intensified draining of lakes and wetlands to increase agricultural land in Sweden from the mid–1800s until the 1950s^69^. However, the modern use of fertilizers and agricultural practices since the 1950s intensified eutrophication of the Baltic Sea^70^, which is reflected in our cores with > 5‰ increase of δ^15^N values during the last 50 to 100 years, indicating an increase in nutrient loads from agricultural activities and the intensification of land-use^48,49^. The increased eutrophication during the 1950s to 1970s has also been observed in coastal sediments of Västervik in previous studies^71^. The change in δ^15^N also marked a decrease in OC and TN content in the Västervik sites since the 1960s, which was less pronounced in the seagrass and unvegetated sites in S:t Anna likely due to their more offshore location. Though, the mixed meadow in S:t Anna showed an increase in δ^13^C (from –19.5 to –20.7) around ~ 1800 CE to present, indicating an earlier change in source composition towards more terrestrial input. This site is in closer proximity to the coast in relation to the other sites in S:t Anna, and the shift in δ^13^C could be related to the onset of increased eutrophication from more intensified land-use, which has been identified to occur around 1800 CE in this area^72^. An increase in δ^15^N was also present in the mixed site from the ~ 1960s, however this did not influence the OC and TN content. The decrease in OC and TN in Västervik could be related to increased land-use with higher contribution of organic matter from cultivated land to the sediment, which is comprised of labile and more easily degradable organic matter in relation to the seagrass plants^73,74^, and a higher input of labile organic matter could also stimulate decomposition of recalcitrant organic matter^75^. This was further indicated by a decrease in δ^13^C-values with about 0.5‰ from the 1960s to present. This highlights that even though positive relationships were found between land-use and OC and TN accumulation over millennial timescales, overly intensified land-use and agricultural activity could have negative consequences for seagrass carbon and nitrogen sinks^45,76^ due to changes in organic matter source composition, degradation potential and carbon remineralization^77^.

### Coastal change

The coastal zones in cold-temperate regions of the Northern Hemisphere have been largely shaped by isostatic land rebound since the last glacial maximum, with clear changes in land uplift in our study sites that resulted in increasing land area over time (with as much as an 80-fold increase in S:t Anna). This increase in land surface created extended hydrodynamically sheltered and shallow areas facilitating seagrass growth, and more potential sources of terrestrial runoff. As lignin is present in both terrestrial vascular plants and the seagrass plants themselves, the increase in lignin concentrations following land uplift could be of both terrigenous and seagrass origin. However, the positive correlations between δ^13^C values and tr4pp, lignin and phenols support the hypothesis that land uplift created more favorable conditions for seagrass meadow productivity and biomass build-up rather than increasing the transport of terrigenous lignin products from land runoff. Terrestrial organic matter in the coastal Baltic Sea shows δ^13^C values ranging from –29 to –22‰^78,79^, whereas tr4pp, lignin and phenols have more less negative values (about –21 to –19‰) associated to marine sources. Furthermore, given the intensified land-use over time in the study areas and the fact that cultivated soils mostly comprise of degraded organic matter with low lignin content^74,80^, this further strengthens the notion that the lignin is of seagrass origin. However, this does not exclude the input of organic matter from land, as seagrass meadows accumulate both allochthonous and autochthonous material^81^. Allochthonous organic matter input ranges from 5 to 90% in *Z. marina* sediments^15^, and the sedimentary OC and TN in the seagrass meadows is likely derived from both the internal seagrass production and multiple allochthonous sources. As *Z. marina* OC accumulation depends to a various degree of sediment supply^82,83^, higher seagrass biomass and canopy height, gained from more favorable conditions, can additionally not only increase the import through particle trapping but also reduce the export of organic matter through sediment stabilization and prevention of erosion^45,84^, and by that further contribute to OC and TN accumulation.

### Seagrass establishment

Based on the appearance of the lignin product tr4pp in the sediment record together with less negative δ^13^C values, the onset of seagrass colonization could be identified in the *Z. marina* meadow in Västervik, which likely occurred around ~ 350 CE, while in the mixed meadow in S:t Anna, the vegetation establishment occurred > 2000 BCE. For the monospecific *Z. marina* meadow in S:t Anna, the seagrass colonization period likely occurred during the last centuries based on the shallow depth of the seagrass-derived sediment (10–13 cm) and given the present sediment accumulation rate. Although the exact time period of seagrass establishment could not be established in the *Z. marina* meadow at S:t Anna, the CPM showed a shift in the sediment biogeochemical properties at this sediment depth and the sediment characteristics were similar to the seagrass site in Västervik, indicating the presence of seagrass. The more recent establishment in the monospecific meadow in S:t Anna is likely due to the offshore location with a relatively late formation of the surrounding sheltered islands and islets to protect from hydrodynamic forces. This is also indicated by an apparent hiatus in the sediment record originating from erosion. The variation in timing of the seagrass establishment in the mixed meadow in S:t Anna and the monospecific meadow in Västervik may be explained by differences in hydrodynamics related to land-uplift and site-specific sedimentation processes as the low DBD and high silt–clay fraction in the sediment profile of the mixed meadow in S:t Anna indicates a stable environment for a longer period of time compared to the seagrass meadow in Västervik. However, as the mixed meadow had been vegetated throughout the time period assessed, the consistency of the sediment biogeochemical properties might be derived from the stabilizing effect of the vegetation.

Prior to the seagrass establishment, there were changes in the biogeochemical characteristics and elemental composition of the sediment in the *Z. marina* meadows in S:t Anna and Västervik (Fig. S5). The changes in elemental composition showed a shift from more lithogenic to organic substrate (reflected by, e.g., a decrease in strontium, potassium and silicate and an increase in bromine, chlorine, nickel and copper)^85^. This was confirmed by the strong factor loadings in PC1 with negative correlation values in the organic matter elements associated to the seagrass-derived sediment in the top of the cores and positive relationships to lithogenic elements at the bottom of the sediment profiles (Fig. S5). Following seagrass establishment, the more organically rich seagrass sediment had as much as 7– to 14–fold increases in OC and TN content, respectively, and a general increase in δ^13^C values. The OC and TN increases were likely due to reduced hydrodynamic forces increasing the entrapment of particles within the seagrass canopy^86,87^ as well as higher preservation with increased lignin content of the seagrass detritus. In the mixed meadow, there were increases in OC, TN, δ^13^C, lignin and tr4pp content around 1500 to 1300 BCE, which indicate a shift to organic matter of more marine origin and potentially higher seagrass biomass abundance^88^, and enrichment of more recalcitrant organic matter (following a diagenesis of more labile organic carbon, which increases the δ^13^C-values)^89,90^. This period (~ 1700 to 1200 BCE) showed more variable climate and a generally wetter and colder environment in Sweden^91^, which potentially could have influenced the shift in seagrass abundance, although the link between climatic variability and seagrass abundance is not clear and the interpretation remains tentative. The shift in OC source input (with less negative δ^13^C-values) and an almost doubling of OC and TN content shows the relationship between increased seagrass biomass and the efficiency of the carbon and nitrogen sink function in coastal environments. Seagrass biomass is mainly composed of carbohydrates, lignin and lignin-derived phenol products^92^. In seagrass sediment, carbohydrates tend to decrease with age while lignin and lignin-derived phenol products are more stable^59^. Phenol products have been seen to increase with age with a selective preservation during diagenesis of lignin^93^. This suggests that the high concentrations of phenol compounds in our *Z. marina* sediment are likely lignin derivatives with high preservation potential. The clear correlation between OC and TN with both lignin and phenol in seagrass meadows and a lack of relationship with phenol in the unvegetated sediments indicate that the *Z. marina* sediments have a high preservation capacity of OC and TN.

Interestingly, the presence of coarser sediment grain-sizes pre-dated the onset of seagrass establishment, which changed to more fine–grained sediments (with higher silt and clay contents) once the seagrass plants had colonized. The CPM also showed changes in the sediment biogeochemical properties in Västervik prior to seagrass establishment at sediment depths of 21–23 cm (~ 250 to 1700 BCE), which further supports a shift in sedimentation and potentially higher hydrodynamics during this period. The pattern with more sandy sediment prior to the seagrass colonization period has also been observed in seagrass meadows in Skagerrak^19^. It may be that too fine–grained sediments hinder seagrass plant establishment and growth in the initial phase of colonization as high silt and clay fractions (especially silty sediment with low cohesion) likely increases water turbidity and lower light attenuation, which reduce the chances for seagrass survival. This indicates a high importance of specific sediment characteristics for seagrass colonization and somewhat mirrors the experience from current seagrass restoration projects showing the relevance of seagrass properties, including a low silt–clay content, for successful re-colonization and replantation^94,95^.

### Carbon and nitrogen accumulation

The short-term (decades to century) seagrass CAR levels were higher (average ± SD: 16 ± 5 g OC m^−2^ yr^−1^ ) than previous estimates in the Baltic Proper (0.8 –3.9 g OC m^−2^ yr^−1^)^22^, while being in the range of the global median CAR of *Z. marina* (14.6 g OC m^−2^ yr^−1^)^96^. The TN burial rates (average ± SD: 1.8 ± 0.5 TN m^−2^ yr^−1^) were similar to the range of *Zostera* spp. accumulation (0.6–4.5 g TN m^−2^ yr^−1^)^17,97^. However, a few studies reported accumulation rates above 120 g OC m^−2^ yr^−1^ and 14 g TN m^−2^ yr^−1^^3,17^. In our study, the short- and long-term accumulation rates of OC and TN were consistently higher in seagrass meadows than unvegetated sites except for the mixed meadow, which showed overall the lowest accumulation due to a low MAR, OC and TN content. The monospecific meadows in S:t Anna and Västervik had similar short-term CAR (20.1 to 21.0 g OC m^−2^ yr^−1^) and NAR (2.0 to 2.5 g TN m^−2^ yr^−1^) despite showing differences in time of seagrass establishment and depth of the seagrass-derived sediment. This is due to the higher MAR and lower OC and TN in S:t Anna with an opposite pattern seen in Västervik, showing that the relative differences in sediment properties can yield similar CAR and NAR levels. This also highlights that short-term CAR and NAR might not be related to the age of the seagrass meadow. However, due to the millennial accumulation of OC and TN in the seagrass meadow in Västervik, the total OC and TN stocks derived from the seagrass meadows are sustainably higher.

The long-term (millennial) OC and TN accumulation rates were up to 8– and tenfold higher than previously measured in *Z. marina* sediment^19^. The stability and permanence of the carbon and nitrogen stocks are dependent on several abiotic and biotic factors, including carbon quality, sediment properties and seagrass disturbances^98^ and preservation of the organic matter was likely due to the presence of seagrass-derived lignin products. The stability of the C/N ratio over time also supports the hypothesis that the decomposition of organic matter in the sediment was low as nutrient concentrations are to a large degree controlling decomposition rates^99^. This shows that Baltic seagrass meadows can have higher accumulation rates and a higher permanence as OC and TN sinks than previous studies have shown^22^ and that mixed seagrass meadows (including *Z. marina*, *Ceratophyllum* sp., *Myriophyllum* sp., *Stuckenia* sp. and *Zannichellia* sp*.*) also hold promise for OC and TN accumulation^78,100^. As other rooted vegetation besides seagrass are common in the Baltic Proper^20,101^, where they occupy a large distribution range^102^, and given these results, these submerged plant communities deserve further research as potential blue carbon habitats. Short- and long-term CAR in *Z. marina* meadows of the Baltic Sea are, however, still lower than global seagrass averages (short-term: 41 ± 7 g OC m^−2^ yr^−1^; long-term: 25 ± 5 g OC m^−2^ yr^−1^)^103^. The assessments of global averages include several species known to have high carbon accumulation rate (e.g., *Posidonia oceanica*)^104^, but the lower CAR levels observed in the Baltic Sea are likely due to the unique environmental conditions (e.g., low salinity, temperature and light attenuation) and seagrass plant adaptations and morphology (e.g., low shoot height and seasonal leaf shedding).

### Sediment carbon and nitrogen storage

The OC and TN (with a maximum of 12.5% OC and 1.2% TN) were substantially higher than previous estimates in the Baltic *Z. marina* meadows, ranging from 0.03 to 1.89% OC and from 0.01 to 0.19% TN^23,25,26,105,106^. In the Baltic Sea, *Z. marina* tends to grow in hydrodynamically exposed areas^107,108^ and several of the previous studies have assessed OC stocks in these environments^25,26^, while the seagrass meadows in this study were found in environments of relatively low hydrodynamic exposure. The lowest %OC and TN were measured in the seagrass and unvegetated sites of S:t Anna, which had the highest relative fetch of the studied sites. This shows the well-documented importance of hydrodynamics as environmental driver for OC and TN accumulation^17,67,109^, but also that *Z. marina* meadows can grow in hydrodynamically more sheltered environments in the Baltic Sea, which in this study was confirmed by the low effective fetch (km), ranging from 0.52 to 0.78^110^. The unvegetated sites showed highly similar sediment biogeochemical properties and accumulation patterns to the adjacent seagrass meadows, supporting the hypothesis that environmental conditions, coastal geomorphology and sedimentation patterns are important drivers for the OC and TN sink capacity in coastal areas^19^. Despite this, unvegetated sediments had significantly lower OC and TN content in comparison to seagrass meadows, which highlights the additional contribution of coastal vegetation for climate change mitigation and nutrient filtration. However, the unvegetated sites showed similar tr4pp concentrations and patterns as the seagrass sites. Another explanation, besides the export of seagrass matter to unvegetated sites, is that the current unvegetated site could have been colonized by seagrass in the past. The higher concentrations of tr4pp in surface sediment of the deep coastal accumulation basin site, however, strengthen the explanation that seagrass-derived organic matter can be transported from surrounding seagrass meadows to adjacent unvegetated sites. This is because the site is more than twice of the maximum depth limit for seagrass plant growth in the Baltic Proper, which is around 7 m^108^, and could therefore not sustain seagrass photosynthesis. However, historically, due to less eutrophication and higher water clarity^21^, the depth limit for seagrass growth in other parts of the Baltic Sea (Kattegat) has been recorded down to 15–20 m depth about 100 years ago^111^, and given the current sea level rise, it is theoretically possible that this site could also have been colonized by seagrass in the past. Therefore, we cannot fully exclude the explanation that currently unvegetated sites could have historically been vegetated.

### The influence of future climate warming on Baltic seagrass systems and ecosystem services

Sea level rise in the studied areas may reach 0.3–0.45 m under a “high-end” emission scenario (RCP 8.5) or 0.05–0.20 m under a “medium” emission scenario (RCP 4.5) (corrected for the local glacial isostatic adjustment) by 2100^112^. As land uplift clearly had a positive effect on the OC and TN accumulation, through creating suitable, sheltered habitats, a future sea level rise may submerge shallow coastal areas and increase hydrodynamic exposure of the seagrass meadows and reduced light attenuation for the seagrass plants. This may reduce the probability of seagrass colonization in the future by uprooting of seedlings and by delimiting potential new areas for colonization. An increased water depth may also decrease survival (in the deeper parts) through limiting photosynthesis. Expected increases in precipitation^113^, especially extreme events, leading to enhanced sedimentation could lead to increased turbidity and lower salinity, which would similarly influence the seagrass survival negatively. Furthermore, extreme sea level events, e.g., related to storms, with historically long (centennial) return periods will occur much more frequently^114^, where short-term flooding and intense wave and wind action could lead to sedimentary carbon and nitrogen erosion and loss of seagrass meadows^115–117^. Finally, Baltic marine heatwaves^118^ will further increase in a warmer climate^119^ and could potentially have negative impacts on the long-term population persistence of *Z. marina*^120^. However, the production and growth of northern populations of *Z. marina*, such as in the Baltic Sea, will likely withstand (and even benefit) from increasing temperature^121^. However, sudden increase in temperatures following a heatwave can lead to thermal stress and *Z. marina* decline^122^. Marine heatwaves have been shown to lead to large dieback of seagrass and erosion of carbon stocks^123,124^.

## Conclusion

In this study, we show that historic land-use and coastal geomorphological changes have influenced the OC and TN sink capacity in coastal sediments. We could identify the occurrence of seagrass colonization in both monospecific meadows, and once the seagrass meadows established, the seagrass plants transformed the biogeochemical properties of the sediment leading to increased accumulation rates of OC and TN through accrual of seagrass plant detritus in the sediment and by trapping of organic matter from land (and other habitats). The colonization of seagrass likely also increased the accumulation of OC and TN in the surrounding habitats as we found seagrass-derived organic matter in adjacent unvegetated habitats, which shows that establishment of seagrass meadows can increase climate change mitigation and nutrient filtration capacity beyond the habitat boundaries. Consequently, this highlights the need to assess and manage coastal carbon and nitrogen sinks at landscape levels. Furthermore, we found that mixed meadows in the Baltic Sea, where the seagrass *Z. marina* and other rooted vegetation (such as *Ceratophyllum* sp., *Myriophyllum* sp., *Stuckenia* sp. and *Zannichellia* sp*.*) co-occur, show promise as blue carbon habitats. Future sea level rise in combination with high intensity human land-use can, however, negatively affect the carbon and nitrogen sink function of seagrass meadows by increasing hydrodynamic exposure and altering the organic matter source composition, which leads to accumulation of less refractory and more degradable organic matter from agricultural sources. Management of Baltic coastal blue carbon habitats, such as seagrass meadows, would benefit from considering landscape scale processes, land-based activities and potential climate change impacts when assessing potential risks and designing protected areas or identifying restoration sites. This requires more adaptive management strategies and mitigation of impacts beyond e.g. the boundaries of a protected area to reduce pressures on both local and regional scales. The findings from this study highlights the importance of integrated seagrass protection and conservation measures, aligned with thorough land-use planning and climate-change mitigation and adaptation strategies, to secure thousands of years of carbon and nitrogen storage.

## Methods

### Study area and sediment core sampling

Sediment cores were collected from two locations, S:t Anna (58°20′49″N, 16°53′57″E) and Västervik (57°42′30″N, 16°42′51″E), situated on the Swedish east coast in the Baltic Proper in May 2023 and July 2022, respectively (Fig. 6). The Baltic Sea is a brackish water system with a salinity gradient ranging from 2 in the northern part (Bothnian Bay) to 25 in the southwest (Kattegat and the Belt Sea) with the salinity in the study locations being around 6 to 7. Due to the salinity gradient, the northern distribution of *Z. marina* is limited to the northern Baltic Proper^102^ as *Z. marina* plants can only sustain salinities down to about 5–6^108^. In S:t Anna, four sites were sampled, including one monospecific *Z. marina* meadow, one mixed *Z. marina* meadow (mixed with other rooted vegetation including *Ceratophyllum* sp., *Myriophyllum* sp., *Stuckenia* sp. and *Z. palustris*) and one unvegetated site, as well as a reference site in a deep coastal accumulation basin (17 m) in Bråviken to assess the contribution of seagrass-derived organic matter input to deeper areas of the coastal zone (Fig. 1). The monospecific *Z. marina* meadow was located the furthest away from the shoreline on the sheltered side of a small island with a low relative fetch of 0.78 km and the core was sampled at a water depth of 5 m, while the mixed *Z. marina* meadow was situated in a sheltered embayment at 3 m water depth and with an even lower relative fetch (0.62 km). The unvegetated sediment was sampled in-between two small islands at a water depth of 6 m and with the lowest relative fetch (0.52 km) in S:t Anna. In Västervik, two sites were sampled, including one monospecific *Z. marina* meadow and one unvegetated site. The two sites were situated in the same embayment at approximately 40 m apart. The *Z. marina* core was located at a water depth of 5.4 m and had a low relative fetch of 0.52 km, and the unvegetated core at 7.3 m water depth and with a low relative fetch (0.64 km) as well. The relative fetch was calculated following the protocol of Rogala^125^. At each site, a total of three cores, approximately 10 m apart, were sampled using 2 m PVC sediment cores. In all *Z. marina* meadows, the sediment core sampling was done in the interior of the meadow to avoid the influence of edge-effects on the sediment properties^126^. Compression of the sediment was assessed once by measuring the inner and outer lengths of the cores when pushed into the sediment. Based on the measurements, a compression factor was calculated, following the equation in Howard et al.^127^, and used to correct the sediment lengths and dry bulk density (DBD) measurements (the compression ranged from 0 to 24%). The cores were opened lengthwise and sliced at 1 cm-thick intervals down to 30 cm, and at 2 cm-thick intervals for the remaining of the core lengths. Before slicing the cores, one half of each core was scanned using an ITRAX X‐ray fluorescence (XRF) core scanner.

### Sediment chronology

To obtain age models for the sediment profiles, concentrations of ^210^Pb were assessed by measuring the decay product (^210^Po) in equilibrium using alpha spectrometry^128^. In each core, concentrations of ^226^Ra were measured in three evenly distributed slices based on its decay product (^214^Pb) using gamma spectrometry. The concentrations of excess ^210^Pb were calculated from the difference between total ^210^Pb and the average ^226^Ra to determine the sedimentation rates and build the age models for the last decades. Radiocarbon (^14^C) dates were obtained along the sediment cores (Table S1), except for the monospecific *Z. marina* meadow and unvegetated site in S:t Anna. In the monospecific *Z. marina* and unvegetated sites in S:t Anna, coarser grain sizes and fluctuations in DBD at 10 to 13 cm indicate a hydrodynamic depositional environment and a potential hiatus in the sediment records from erosion as post-glacial clays were present at shallow sediment depths (Fig. S2), and therefore no radiocarbon dating was performed. We assumed that the post-glacial clays were derived from the retreat of the Scandinavian Ice Sheet and hence being of a much older age (i.e., early Holocene). In each of the other cores, 3–5 ^14^C age values were obtained (Table S1). The Marine20 calibration curve^129^ was used to calibrate the ^14^C ages and we applied a local marine reservoir correction (ΔR = –294 ± 50 years) derived from the Marine20 reservoir database (map no. 1710). Sediment depth-age models based on the ^210^Pb-derived ages (using the CRS model^130^) and calibrated ^14^C ages were calculated using the Bacon R-package^131^. Sediment accumulation rates (SAR) and mass accumulation rates (MAR) were calculated using a CF:CS (Constant Flux:Constant Sedimentation) model for the last decades based on ^210^Pb^132^^,^^133^, and using the Bacon model for the deeper parts of the sediment profiles. Three radiocarbon dates from carbonate shells and plants were omitted from the models (i.e. two from the *Z. marina* meadow in Västervik and one from the mixed *Z. marina* meadow in S:t Anna) because of inversed ages (Table S1).

### Sediment biogeochemical analysis

Sediment slices were dried at 60 °C until constant weight to estimate dry bulk density (DBD, g cm^-3^). For organic carbon (OC) and total nitrogen (TN) contents and C and N stable isotopic composition (δ^13^C and δ^15^N), sediment slices from 0 to 10 cm and subsequently for each 5 cm until the end of the core were analyzed using a Flash EA 1112 Series Elemental Analyzer connected to a Conflo III and a DeltaV Advantage Isotope Ratio Mass Spectrometer (Thermo Finnigan, Bremen, Germany). The isotopic compositions were expressed in the delta notation relative to VPDB (Vienna PeeDee Belemnite) for δ^13^C and to atmospheric nitrogen standard for δ^15^N. For reference material, Caffeine IAEA-600 (δ^13^C =  − 27.77 ± 0.04‰, δ^15^N = 1.00 ± 0.20‰), Potassium Nitrate IAEA-NO-3 (δ^15^N = 4.7 ± 0.2‰) and Graphite USGS24 (δ^13^C =  − 16.05 ± 0.04‰) were used. These standards were run for every 12 samples and had a standard deviation of less than 0.08‰ for carbon and 0.2‰ for nitrogen. Prior to OC and TN measurements, the sediment samples were ground into a fine powder using mortar and pestle. Each sample was run in duplicate, with the sample for measuring OC content and δ^13^C treated with 1 M HCl for the removal of inorganic carbon (direct addition using a pipet)^25^, while the other duplicate sample was used for measuring TN content and δ^15^N without pre-treatment. The carbon accumulation rates (CAR) and nitrogen accumulation rates (NAR) were calculated based on the weighted mean of OC and N contents multiplied with MAR.

Grain size distribution was performed using a laser diffraction particle size analyzer (Mastersizer 2000 MALVERN) and calculated as proportions of the different fractions following the classification of Wentworth^134^. The samples were sieved by 2 mm and the fraction < 2 mm treated with 15 to 30% H₂O₂ (depending on the organic carbon content) to remove organic matter prior to analysis. After the analysis, the proportions of fractions were adjusted considering the weight of the fraction > 2 mm.

The preparation of samples for Py-GC–MS analysis followed the protocol of Kaal et al.^55^. Prior to Py-GC–MS analysis, organic matter was concentrated and carbonates and reactive minerals removed using a mild HCl/HF treatment. Each sediment sample (0.4 g) was weighed in 50 mL polypropylene tubes, to which 1 mL of 12 M HCl (aq) was added, followed by dilution with deionized water until the 40 mL mark, centrifugation (5 min at 2500 rpm) and decantation. Thereafter, 5% HF (aq) solution was added until the 40 mL mark, followed by three cycles of washing with deionized water, centrifugation and decantation. The final residue was dried overnight at 50 °C. Pyrolysis-GC–MS was performed at 650 °C (set-point temperature) for 20 s at a heating rate of 10 °C/ms using a Pyroprobe 5000 (CDS Analytical, USA). The interface to the 8860 gas chromatograph (Agilent Technologies, USA) and its injector were held at 325 °C. We used a HP-5MS column, helium as carrier gas, split mode (1:10) and an oven program from 80 °C to 325 °C at 20 °C/min (3 min final hold). The mass spectrometer (5977B, Agilent technologies, USA) scanned in the 50–400 m*/z* range and operated under standard 70 eV EI conditions^55,57^. The pyrolysis product peaks were integrated using their dominant *m/z* fragment and relative proportions were determined as the % of total quantified peak area. The tr4pp, which is a phenolic compound that is relatively abundant in seagrasses, including *Z. marina* pyrolyzates, was used here as a proxy of seagrass inputs^55–57^, together with the sum of lignin products. Terrestrial runoff can also deliver some of the lignin signal to the sediment (but local plankton communities from the water column or microbial communities in the sediment cannot generate this fingerprint).

XRF measurements were analyzed using an ITRAX XRF core scanner (Cox Analytical Systems) to obtain μ-XRF elemental profiles (for Si, S, Cl, K, Ca, Ti, Mn, Fe, Ni, Cu, Zn, Br, Rb, Sr, Zr and Pb). For the analysis, the measurements were performed using a Mo tube with the setting of 30 kV and 50 mA, and a step size of 500 μm with a dwell time of 25 s. The μ-XRF elemental profiles were then transformed using a centered log ratio (CLR) transformation to account for changes in the physical properties along the cores and handling of non-linear effects of the elemental matrix^85,135^.

### Landscape openness data and land uplift spatial modelling

Human-induced landscape openness (i.e., proportions of grassland and cropland) was based on previous analysis from Vinogradova et al., (^64^, unpublished) in which high-resolution pollen stratigraphies were used in regional land cover models (calculated using the Landscape Reconstruction Algorithm; Sugita^136^. Coastal land uplift reconstruction based on eustatic sea level fluctuations and isostatic change was analyzed in ArcGIS pro (v. 3.0). Shoreline placement (m a.s.l) for the last 6,000 years were extracted from local shoreline displacement curves from Katrantsiotis et al.^137^ and Plikk et al.^138^, and coastal geomorphology changes were calculated from digital elevation models (with a 0.1 altitude resolution available from the Swedish National Land Survey) using the raster calculator function.

### Statistical analysis

All statistical analyses were performed in R (v. 4.2.2) and the assumptions of normal distribution and homogeneous variances (for the comparison analysis) were assessed prior to analysis. In order to explore similarities in downcore biogeochemical trends, principal component analysis (PCA) was used. The biogeochemical properties included in the PCA analysis were derived from results of the XRF core scanning (K, Ti, Si, Mn, Ca, Fe, Rb, Zn, Sr, Zr, S, Ni, Cl and Br), CN elemental and stable isotope analysis (% OC, % TN, δ^15^N, δ^13^C), grain size analysis (silt–clay content) and DBD. As not all biogeochemical analyses were performed in each sediment layer, the missMDA-package was used to impute missing values in the sediment profiles prior to the PCA analysis. Diagnostic tests in the missMDA-package were used to assess the influence of imputation on the final PCA model and the biogeochemical properties interpreted as over-imputed were deleted from the analysis. Change point modelling (CPM) was used to identify change points of the downcores of PC1 and PC2 scores for each sediment core using the RBeast-package^139^. Linear regression analysis was used to assess the relationship between the PC2 scores and proportion of land use in terms of alterations from forest to open vegetation (including cropland and grassland). Linear regression analysis was also used to explore the correlative relationships between two predictors, i.e., OC and TN contents, and biomolecular compounds (tr4pp, lignin and phenols) as well as between lignin proportions and land area (km^2^) in buffer zones (diameter: 2 km) of the sampling areas. We applied a Generalized Additive Mixed Model (GAMM) to assess the difference between the monospecific seagrass meadows (not including the mixed *Zostera* meadow) and the unvegetated sites using location (i.e., S:t Anna and Västervik) as a random factor.

## Supplementary Information

Below is the link to the electronic supplementary material.

Supplementary Material 1.

## Data Availability

The dataset used in this study is published on Zenodo (10.5281/zenodo.18754986).

## Declarations

No specific permission was required for the sampled sediment material.
