# Supplementary material for: Coastal land uplift and intensified land-use influence seagrass carbon and nitrogen sink capacity over millennial timescales
Source: Sci Rep. 2026 May 26;16:16263. doi: 10.1038/s41598-026-54674-y (PMC13212900; doi:10.1038/s41598-026-54674-y)
Supplement: Supplementary file 1 — Supplementary Material 1. [file 41598_2026_54674_MOESM1_ESM.docx]

**Supplementary material for Coastal land uplift and intensified land-use influence seagrass carbon and nitrogen sink capacity over millennial timescales**

Martin Dahl^1*^, Sara Braun^1^, Maria E. Asplund^2^, Mats Björk^3^, Joeri Kaal^4^, Hans W. Linderholm^5^, Elinor Andrén^1^, Thomas Andrén^1^, Žilvinas Ežerinskis^6^, Madhavu Vidya Faizal^1^, Sara C. Forsberg^1^, Andrius Garbaras^6^, Malin E. Kylander^7^, Pere Masqué^8,9^, Miguel A. Mateo^8,10^, Justina Šapolaitė^6^, Oscar Serrano^8,10^, J Robin Svensson^11^, Olena Vinogradova^1^, Martin Gullström^1^

^1^School of Natural Sciences, Technology and Environmental Studies, Södertörn University, Huddinge, Sweden

^2^Department of Biological and Environmental Sciences, University of Gothenburg, Kristineberg, Fiskebäckskil, Sweden

^3^Department of Ecology, Environment and Plant Sciences, Stockholm University, Stockholm, Sweden

^4^Pyrolyscience, Santiago de Compostela, Spain

^5^Regional Climate Group, Department of Earth Sciences, University of Gothenburg, Gothenburg, Sweden

^6^Center for Physical Sciences and Technology, Vilnius, Lithuania

^7^Department of Geological Sciences, Stockholm University, Stockholm, Sweden

^8^Centre for Marine Ecosystems Research, School of Natural Sciences, Edith Cowan University, Joondalup WA, Australia.

^9^International Atomic Energy Agency, Principality of Monaco, Monaco

^10^Centro de Estudios Avanzados de Blanes, Consejo Superior de Investigaciones Científicas (CEAB-CSIC), Blanes, Spain

^11^Department of Marine Sciences, University of Gothenburg, Gothenburg, Sweden

*Corresponding author: Martin Dahl (martin.dahl@sh.se)

**
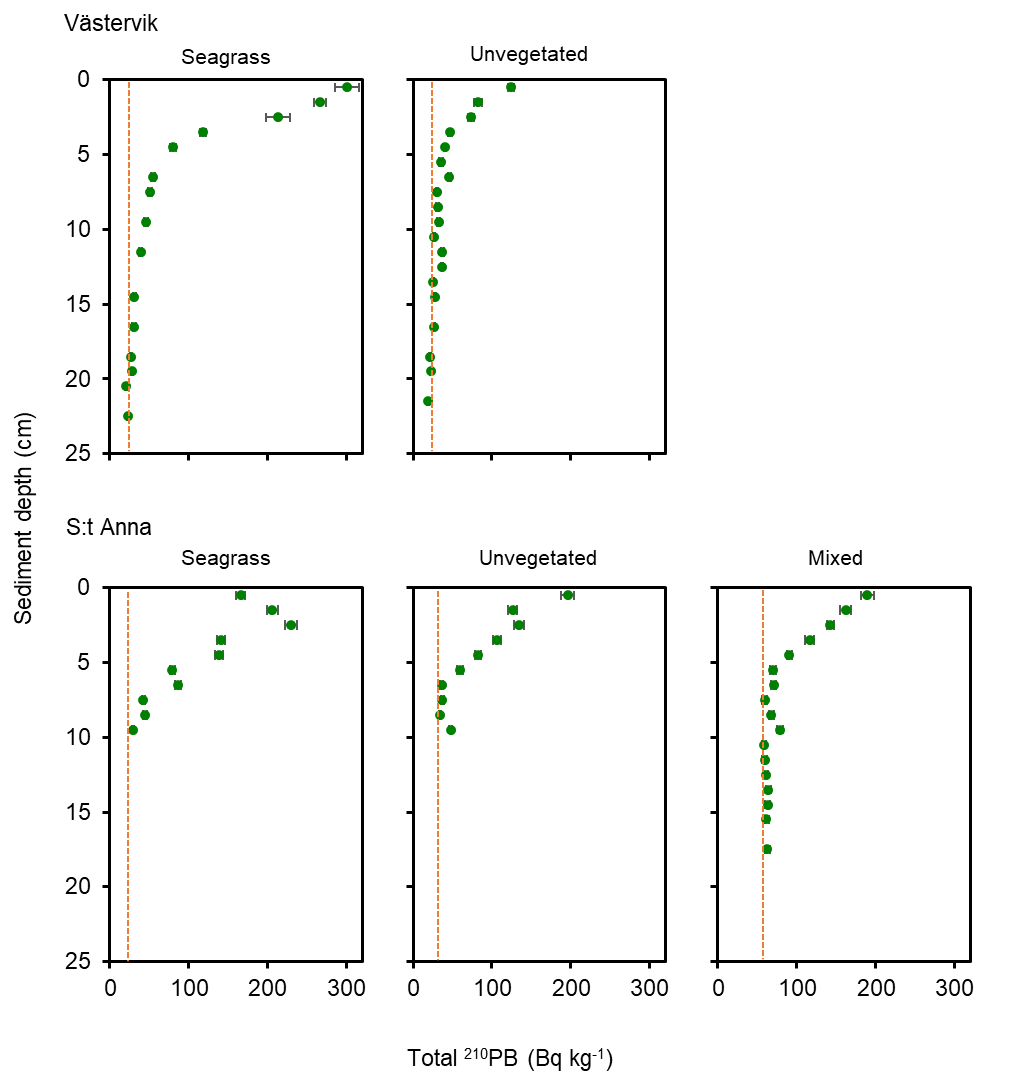
**

Figure S1. Profiles of total ^210^Pb for the sediment cores to estimate short-term ages (~100 years) and sedimentation rates. The dashed orange lines show the supported ^210^Pb levels, from which sedimentation rates can be calculated.


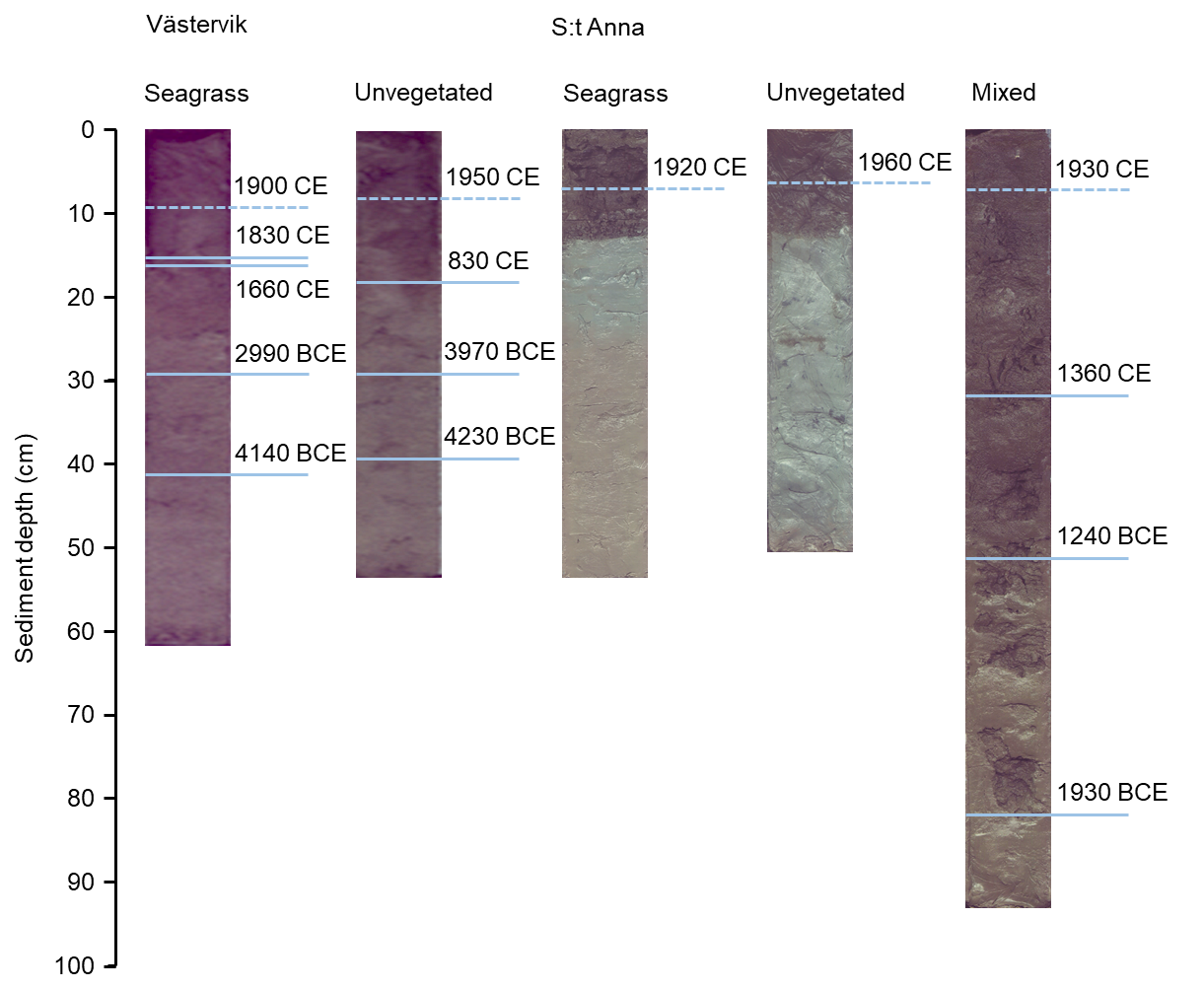


Figure S2. Scanned imagery of sediment cores. The lines show the layers for ^210^Pb (dashed lines) and calibrated ^14^C dates (solid lines).


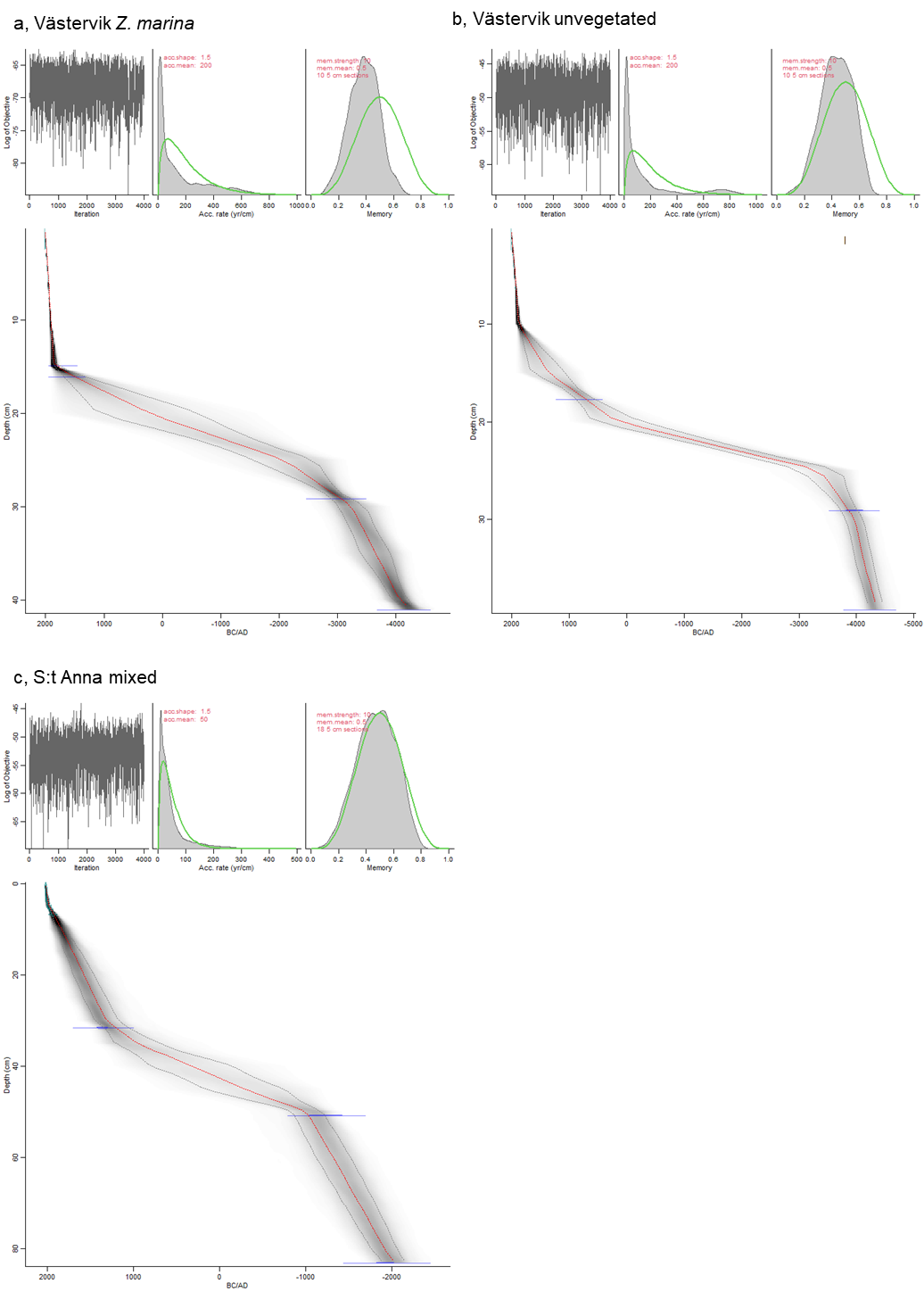


Figure S3. Age-depth models for the *Z. marina* (A) and unvegetated (B) sites in Västervik and the mixed site in S:t Anna (C). The solid red lines indicate the best “fitted” model based on ^210^Pb-derived ages and calibrated ^14^C-dates and the dashed lines are 2 σ range (equal to 0.95) of the model. The blue horizontal lines show the calibrated ^14^C dates (with 2 σ range).


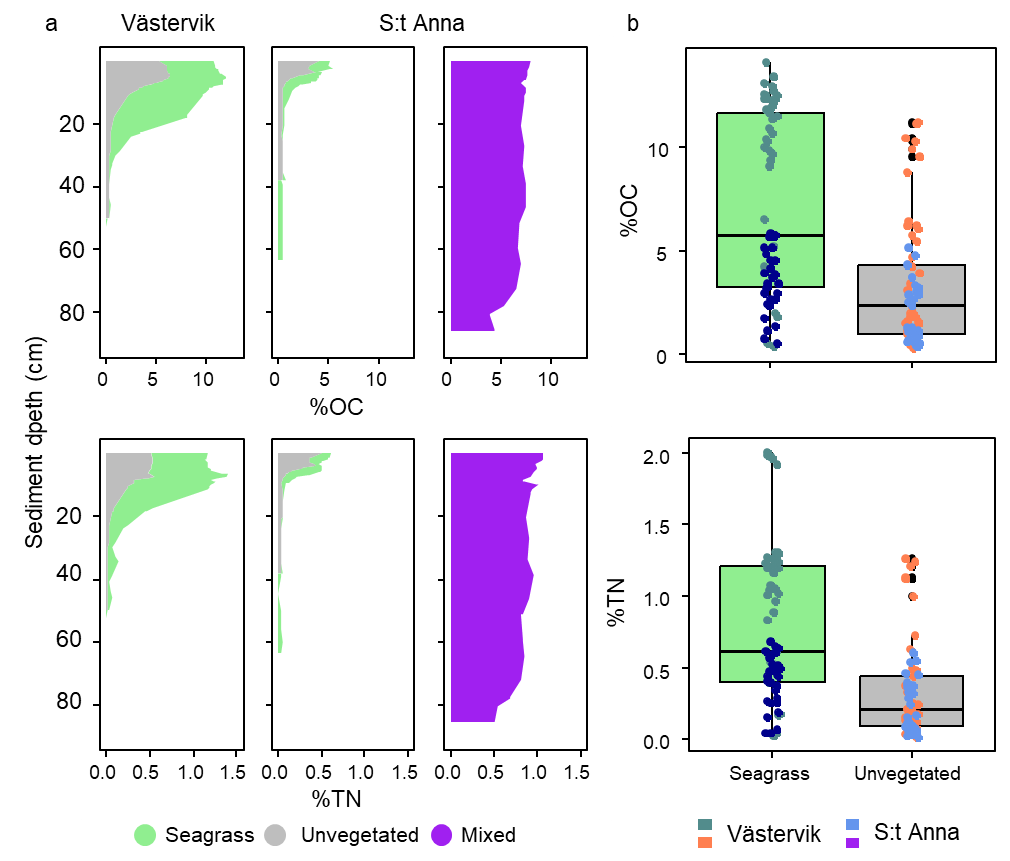


Figure S4. The organic carbon (OC) and nitrogen (TN) content in seagrass, unvegetated and mixed meadow sediments (A) and the statistical comparison of organic carbon (OC) and nitrogen (TN) contents between seagrass (excluding the mixed *Z. marina* meadow in S:t Anna) and unvegetated sediments (n = 3 sediment cores per habitat) (B). The lines in the boxes show median values, the box edges represent the 75% and 25% percentiles, and the error bars are 95% confidence intervals. The black dots are outliers.


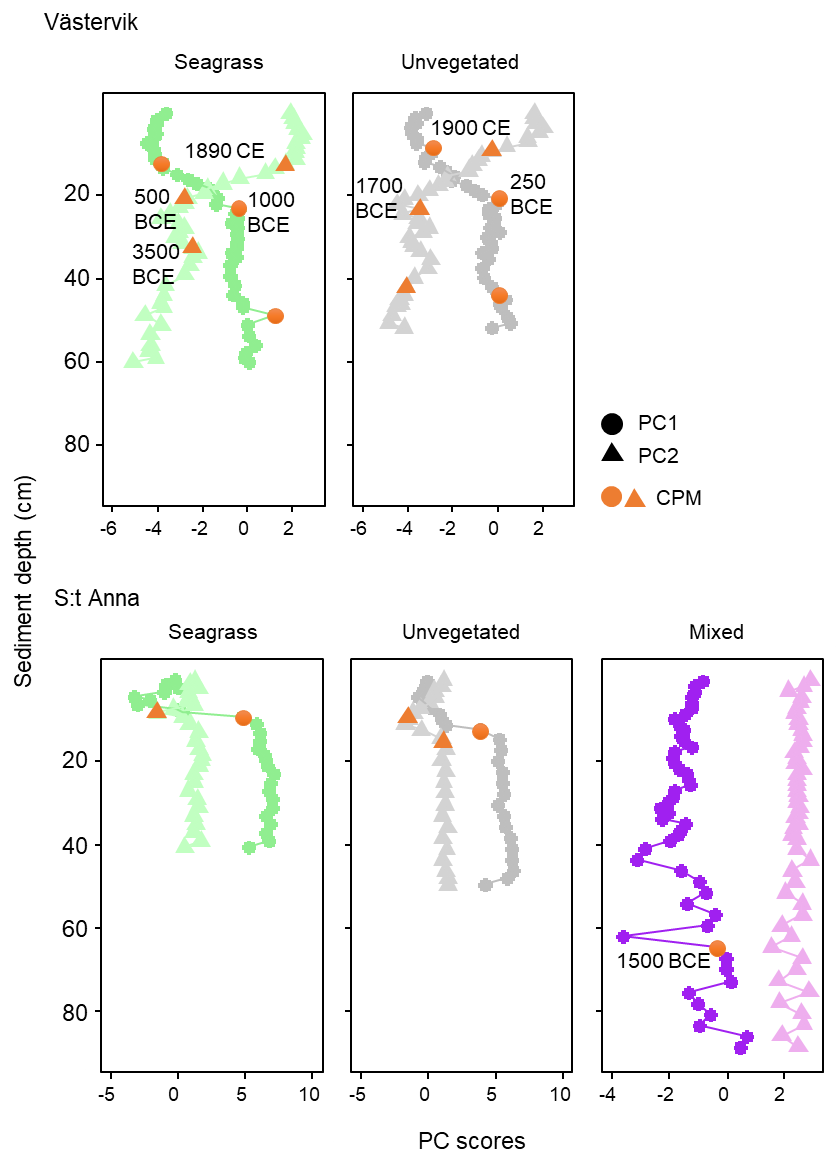


Figure S5. Sediment downcore trends for Principal Components (PC) 1 and 2 scores and estimated breakpoints based on Change Point Modeling (CPM). Note that the seagrass and unvegetated cores in S:t Anna and the two deepest change points in Västervik lack estimated ages as these occurred prior to the maximum age of the age-depth model.

Table S1. Radiocarbon dates (corrected for reservoir effect) used for the sediment age-depth Bacon models in seagrass and unvegetated sites in Västervik and mixed meadow in S:t Anna. BP = before present, BCE = before common era, CE = common era.

| Site | Laboratory code | Material type | Decompressed sediment depth (cm) | ^14^C age (yr BP) | Mean cal. age (yr BP)  (min - max) | Years (BCE/CE) |
| --- | --- | --- | --- | --- | --- | --- |
| Västervik  *Z. marina* | FTMC-VS69-14 | Plant material | 15 | 345 | 117  (-2 - 152) | 1833 CE |
|  | FTMC-VS69-15 | Bulk sediment | 16 | 535 | 289  (209 - 403) | 1661 CE |
|  | FTMC-ZH65-7 | Bulk sediment | 29 | 4549 | 4936  (4822 - 5028) | 2986 BCE |
|  | FTMC-VS69-17 | Bulk sediment | 41 | 5582 | 6093  (5995 - 6190) | 4143 BCE |
|  | FTMC-VS69-18 | Shells | 59 | -227* | - | - |
|  | FTMC-VS69-20 | Shells | 60 | -89* | - | - |
| Västervik unvegetated | FTMC-VS69-11 | Bulk sediment | 18 | 1440 | 1127  (1050 - 1230) | 823 CE |
|  | FTMC-VS69-12 | Bulk sediment | 29 | 5421 | 5921  (5995 - 6190) | 3971 BCE |
|  | FTMC-VS69-13 | Bulk sediment | 39 | 5667 | 6178  (6096 - 6287) | 4228 BCE |
| S:t Anna mixed | FTMC-ZH65-3 | Bulk sediment | 32 | 887 | 592  (525 - 655) | 1358 CE |
|  | FTMC-ZH65-4 | Bulk sediment | 51 | 3193 | 3194  (3100 - 3310) | 1244 BCE |
|  | FTMC-ZH65-5 | Plant material | 76 | -279* | - | - |
|  | FTMC-ZH65-6 | Bulk sediment | 83 | 3745 | 3881  (3763 - 3990) | 1931 BCE |

^*^Not included in the age-depth models or calculated as calibrated ages due to inversed ages.

Table S2. The average (±SD) carbon and total nitrogen (C:N) ratio, % organic carbon (OC) and % total nitrogen (TN) for three different time intervals, i.e., short-term based on estimates from ^210^Pb dating (~100 years, between 75 and 125 years), long term (~1000 years) and since the colonization of seagrass based on age estimates from the Bacon age-depth models.

|  |  | Time interval | C:N | % OC | % TN |
| --- | --- | --- | --- | --- | --- |
| Västervik | Seagrass | Short term | 10.1 ± 0.3 | 12.5 ± 0.6 | 1.2 ± 0.04 |
|  |  | Long term | 10.3 ± 0.4 | 9.8 ± 4.8 | 1.0 ± 0.5 |
|  |  | Seagrass colonization | 10.3 ± 0.4 | 9.3 ± 5.0 | 0.9 ± 0.5 |
|  | Unvegetated | Short term | 9.5 ± 2.0 | 10.2 ± 0.9 | 1.1 ± 0.2 |
|  |  | Long term | 9.6 ± 1.8 | 6.5 ± 3.8 | 0.7 ± 0.4 |
| S:t Anna | Seagrass | Short term | 8.5 ± 0.6 | 5.1 ± 0.8 | 0.6 ± 0.07 |
|  | Unvegetated | Short term | 8.3 ± 0.5 | 2.7 ± 1.3 | 0.3 ± 0.2 |
|  | Mixed | Short term | 7.7 ± 0.2 | 7.4 ± 0.1 | 1.0 ± 0.03 |
|  |  | Long term | 7.9 ± 0.2 | 7.5 ± 0.2 | 1.0 ± 0.03 |
